# Fc γ receptor compositional heterogeneity: Considerations for immunotherapy development

**DOI:** 10.1074/jbc.REV120.013168

**Published:** 2020-11-22

**Authors:** Adam W. Barb

**Affiliations:** Department of Biochemistry and Molecular Biology, Complex Carbohydrate Research Center, University of Georgia, Athens, Georgia, USA

**Keywords:** Fc receptor, antibody, immunotherapy, glycobiology, glycoprotein, Fc, crystallizable fragment, FcγRs, Fc γ receptors, IgG, immunoglobulin G, LacNAc, N-acetyllactosamine, MHC, major histocompatibility complex, NK, natural killer

## Abstract

The antibody-binding crystallizable fragment (Fc) γ receptors (FcγRs) are expressed by leukocytes and activate or suppress a cellular response once engaged with an antibody-coated target. Therapeutic mAbs that require FcγR binding for therapeutic efficacy are now frontline treatments for multiple diseases. However, substantially fewer development efforts are focused on the FcγRs, despite accounting for half of the antibody–receptor complex. The recent success of engineered cell-based immunotherapies now provides a mechanism to introduce modified FcγRs into the clinic. FcγRs are highly heterogeneous because of multiple functionally distinct alleles for many genes, the presence of membrane-tethered and soluble forms, and a high degree of post-translational modification, notably asparagine-linked glycans. One significant factor limiting FcγR improvement is the fundamental lack of knowledge regarding endogenous receptor forms present in the human body. This review describes the composition of FcγRs isolated from primary human leukocytes, summarizes recent efforts to engineer FcγRs, and concludes with a description of potential FcγR features to enrich for enhanced function. Further understanding FcγR biology could accelerate the development of new clinical therapies targeting immune-related disease.

## Fc γ receptors as warheads for cell-based immunotherapies

**Figure 1:**
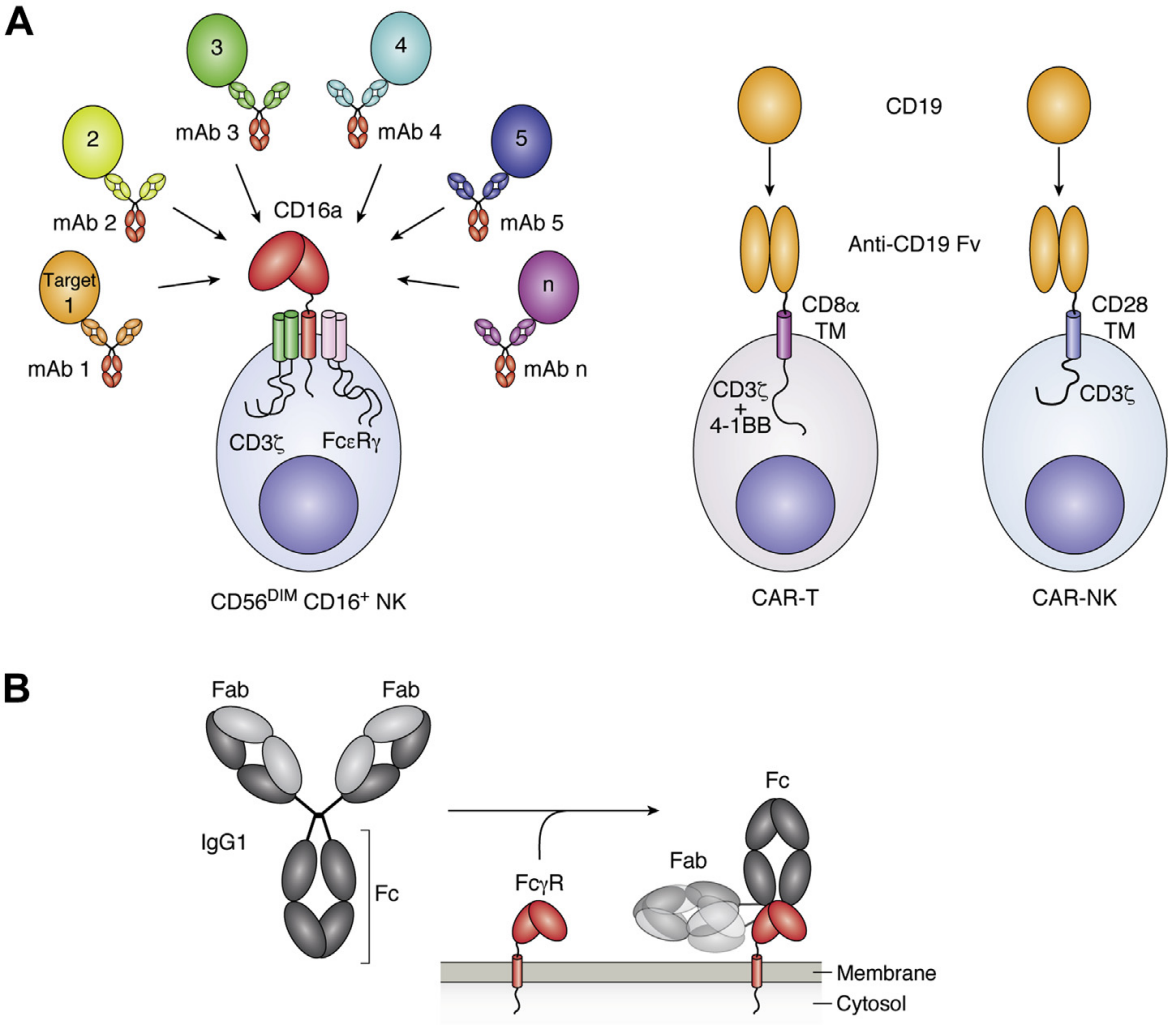
**NK cells naturally target multiple antigens by binding the conserved crystallizable fragment (Fc) of IgG, unlike chimeric antigen receptor (CAR)–T cells or CAR–NK cells that are programmed to recognize a single antigen.***A*, NK cell Fc γ receptor IIIa/CD16a associates with either CD3ζ or the Fc ε receptor γ chain to activate an NK cell after ligation to an antibody-bound target (70, 71). The CAR-T example shown represents the tisagenlecleucel (Kymriah) therapy that targets CD19 on B-cell lymphoma. This CAR–NK construct likewise recently showed success (3, 72). Both CARs contain custom transmembrane and activation domains. *B*, Fc γ receptors bind to the crystallizable fragment of IgG. IgG, immunoglobulin G; NK, natural killer.

Crystallizable fragment (Fc) γ receptors (FcγRs) bind to immunoglobulin G (IgG) antibodies at the surface of a white blood cell (leukocyte) and are required for the efficacy of many antibody-based drugs used to treat diseases (termed therapeutic mAbs). Thus, FcγRs link the target-binding specificity of antibodies to the cytotoxic properties of leukocytes, with an individual FcγR type contributing to the treatment of multiple diseases (Fig. 1). In general, the FcγRs bind the IgG1 and IgG3 subclasses with greater affinity but show lower or negligible affinity for IgG2 and IgG4 (1). The rapid proliferation of mAbs of mostly the IgG1 subclass has focused on binding new targets to treat different diseases and more recently enhancing the FcγR-binding affinity to improve efficacy. Although mAbs are currently drugs, it is theoretically possible to improve affinity by engineering either the antibody or the FcγR. Creating drugs from engineered FcγRs was previously impractical because of the lack of appropriate cell-based therapies, but recent advances in engineered leukocyte therapies provide a vehicle to deploy modified receptors in the clinic.

The recent cell-based immunotherapy revolution has demonstrated the therapeutic and commercial viability of genetically modified lymphocytes to combat even late-stage disease. Lymphocytes are a subset of leukocytes that include antibody-producing B cells, T cells that can kill diseased tissue directly or recruit an immune response by activating other cell types, and natural killer (NK) cells that can destroy antibody-coated tissue and other foreign tissues. Chimeric antigen receptor (CAR) T-cell therapies, including Kymriah (tisagenlecleucel) and Yescarta (axicabtagene ciloleucel), treat B-cell lymphoma by reprogramming the patient’s own T cells to express an engineered CD19-binding receptor to destroy cancerous tissue (Fig. 1). CAR T limitations include cost at ∼$400k/treatment, the near-absolute requirement for autologous transplantation (using the patient’s own tissue) to avoid graft *versus* host disease, cytokine release syndrome, and the narrow therapeutic benefit of a single CAR (2). NK cells offer an alternative CAR expression platform that promises rapid deployability and off-the-shelf availability. Furthermore, NK cells also express a potent FcγR at high levels: FcγRIIIa/CD16a. CD16a binds antibodies coated on the surface of a target cell to trigger a cytotoxic NK cell response (Fig. 1*B*), and it is well established that increased antibody binding increases cytotoxicity and the therapeutic efficacy of mAbs (discussed below). Thus, in addition to hosting CARs, NK cells are suitable hosts for engineered FcγRs.

Unlike T cells, allogeneic NK cell transplants (from a genetically mismatched donor) are tolerated and can be developed from cord blood (3) or cultured cells (4, 5) or transferred from donors without matching major histocompatibility complex (MHC) loci (6). This property allows a single NK cell source to be used to treat multiple patients, in contrast to T-cell therapy production, which is individualized because of the strict requirement for MHC matching. Interestingly, autologous NK cell transplants often exhibit limited efficacy because of reduced NK cell function; however, allogeneic transplantation leads to favorable graft *versus* tumor effects. Thus, allogeneic NK cells promise well-tolerated and effective off-the-shelf treatments with reduced cost and side effects.

As indicated above, future lymphocyte-based treatments are poised to expand beyond CAR incorporation to leverage innate NK cell features. NK cells adopt a vital role in surveillance and clearing diseased tissue. NK cell engineering efforts focus on enhancing these natural functions. Multiple NK cell engineering avenues are being pursued, including CAR NK cells with dramatic recent success (3), NK cells with increased expression of FcγRIIIa/CD16a (Artiva Biotherapeutics; clinical trials as a combination therapy planned for 2020), and cultured NK92 cells (13 current food & drug administration-registered trials as of July 2020) including multiple CD16a-expressing variants.

The importance of FcγRs in current immunotherapies is well established as discussed below with multiple contemporary efforts aimed at improving immunotherapies through FcγR engineering, whether at the amino acid level or by increasing the expression of activating FcγRs on leukocytes. This article will focus on recent definitions of the specific FcγR forms found in the human body, many of which are highly variable because of extensive post-translational modification. Each individual receptor form potentially exhibits distinct characteristics, and certain forms may provide substantial therapeutic benefit after enrichment. This article will also summarize recent efforts to improve NK cell function through FcγR engineering at the amino acid level and identify motifs for future FcγR engineering.

## Antibody-binding FcγRs

The canonical FcγRs are expressed on a variety of leukocytes and are subdivided into activating receptors (FcγRI/CD64, FcγRIIa/CD32a, FcγRIIc/CD32c, FcγRIIIa/CD16a, and FcγRIIIb/CD16b) and inhibitory receptors (FcγRIIb/CD32b). These receptors all bind IgG subclasses, however, with different affinities (Table 1 (7, 8)). CD16a is the primary receptor for anticancer mAbs and is the only FcγR expressed on NK cells for 85 to 93% of the population; the remainder express low levels of CD32c (9). CD16a is also expressed by macrophages and some circulating monocytes that adopt a critical role in clearing antibody-coated targets (10). CD16b is a related receptor that is expressed at very high levels on neutrophils, although the role of CD16b is unclear and it may both promote and inhibit a cellular response (11). CD32a is an activating receptor widely expressed on all leukocytes with the exception of T and B lymphocytes. CD32b is similar to CD32a but is mainly expressed on B cells and functions to inhibit B-cell maturation. CD64 is inducible on monocytes, macrophages, neutrophils, and dendritic cells and binds IgG1 with high affinity (∼1 nM), unlike the other receptors that are considered “low affinity” with dissociation constants ranging from low nM to low μM (1, 12).

**Table 1:** Properties of human Fc γ receptors

FcγR	Function	Affinity	Major alleles	N-glycan sites	Expressed by
CD16a/FcγRIIIa	Activation	IgG3>IgG1>>IgG4>IgG2	V158/F158	5	NK, Mon^a^, MΦ^a^, T^a^, Ba^a^, M^a^
CD16b/FcγRIIIb	Activation	IgG3>IgG1	NA1/NA2/SH	4/6/6	N, Ba^a^, E^b^
CD32a/FcγRIIa	Activation	IgG1>IgG3>IgG2=IgG4	H131/R131	2	MΦ, N, P, M, E
CD32b/FcγRIIb	Inhibition	IgG1=IgG3=IgG4>IgG2	na	3	B, MΦ, D, Ba, Mon^a^, N^a^
CD32c/FcγRIIc	Activation	IgG1=IgG3=IgG4>IgG2	na	3	B^a^, NK^a^
CD64/FcγRI	Activation	IgG1=IgG3>IgG4	na	7	MΦ, Mon, D, M, N^b^

B, B cells; Ba, basophils; D, dendritic cells; E, eosinophils; M, mast cells; MΦ, macrophages; Mon, monocytes; N, neutrophils; na, not applicable; NK, natural killer cells; P, platelets; T, T cells.

a Indicates expression in a subset of cells.

b Indicates inducible expression.

It is well established that increasing Fc-mediated binding by activating FcγRs improves cytotoxicity (13, 14). For example, the anti-CD20 mAb Gazyva (obinutuzumab) increased progression-free survival in non-Hodgkin’s lymphoma by 50% over the standard Rituxan (rituximab) therapy (15). Gazyva and Rituxan bind overlapping CD20 epitopes; however, Gazyva binds CD16a with a 7-fold greater affinity (16, 17, 18). Supported by this example and many others, antibody engineering through amino acid substitutions or glycan engineering promises to increase mAb efficacy (19). Alternatively, affinity can likewise be improved through modifying the receptor. One naturally occurring example exists: patients expressing the CD16a allele encoding a Val residue at position 158 as opposed to the more common Phe had binding of IgG1 2- to 4-fold tighter and showed a substantially improved clinical response to mAb therapies (1, 20, 21, 22, 23, 24).

**Figure 2:**
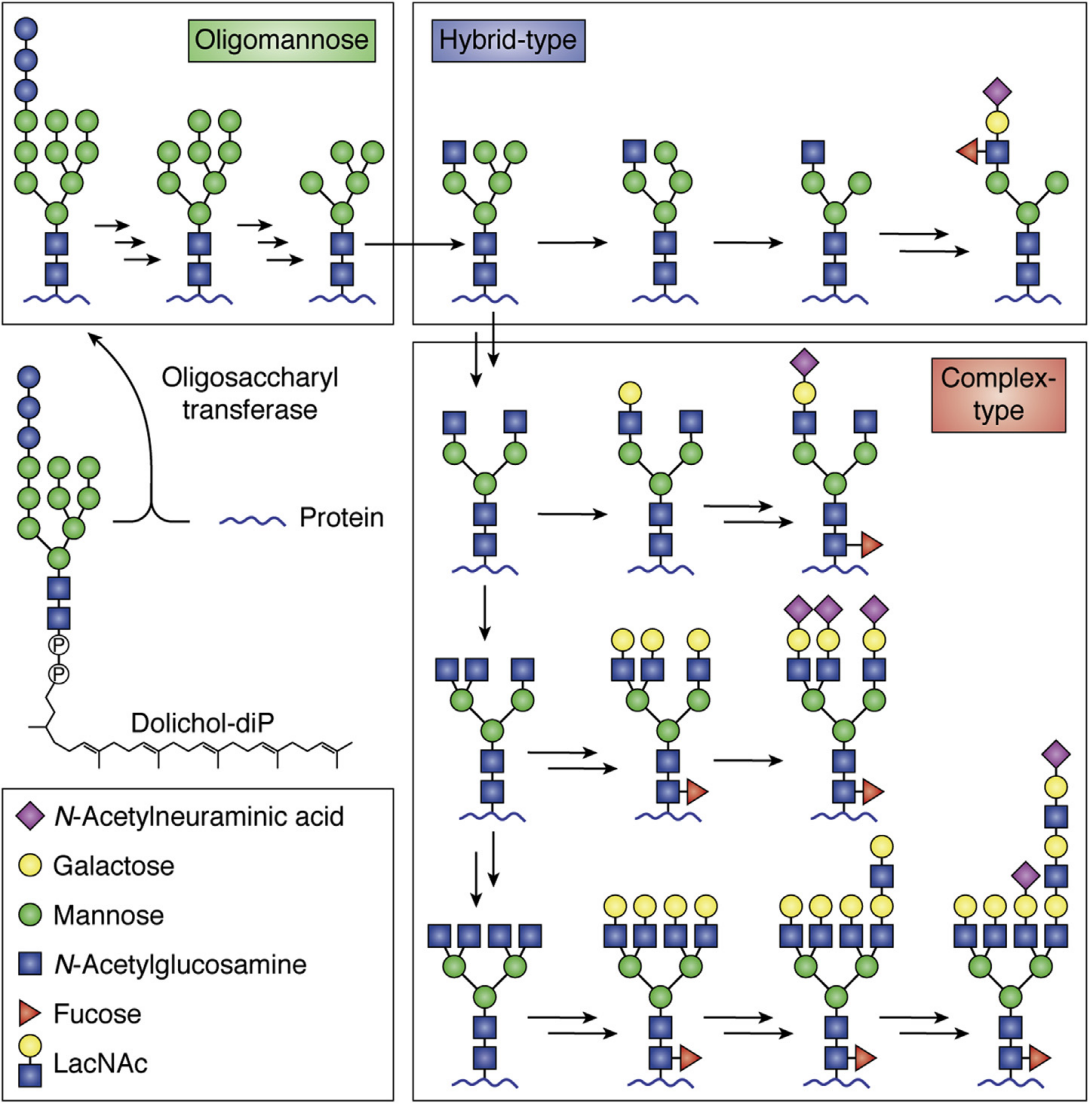
**Simplified mammalian N-glycan processing scheme showing the three main N-glycan types and heterogeneity found on secreted proteins.** Multiple *arrows* indicate multiple processing steps. Note that remodeling reactions occur without a template or proof-reading mechanisms. N-glycan, glycans attached to Asn residues.

One key source of protein heterogeneity is the attachment of complex carbohydrates (glycans) during protein expression and secretion with some glycans extending to dozens of residues. Glycans are common modifications on endoplasmic reticulum, Golgi, and secreted proteins (including serum proteins) that mediate protein folding, oligomerization, stability, and protein function as well as harbor motifs recognized by receptors to name a few key roles although many others exist. Glycans attached to Asn residues (N-glycans) or Ser and Thr residues (O-glycans) are particularly prevalent. N-Glycans are remodeled through extension and trimming reactions in the endoplasmic reticulum and Golgi during protein secretion to form three main classes: minimally remodeled oligomannose forms, hybrid glycans with intermediate processing, and complex types that are heavily modified and often contain terminal N-acetylneuraminic acid caps at the nonreducing branch termini (see Fig. 2 (25)).

FcγR modifications affect antibody-binding affinity and therefore represent a viable engineering target. All FcγRs are heavily modified with N-glycans during protein folding (11). Two groups independently noted that removing sialic acid residues from CD16a increased antibody-binding affinity (26, 27). At the same time, Patel *et al.* (28) reported that changing the composition of CD16a N-glycans from complex to oligomannose types increased antibody-binding affinity by 12-fold. Cambay *et al.* (29) reported a similar result, although with a lower magnitude of less than 2-fold. Further observations indicated that CD16a was the only “low-affinity” FcγR to exhibit this composition-dependent property *in vitro*, and composition of the CD16a N162 glycan primarily affected antibody-binding affinity (30, 31). Although clearly implicating glycan engineering as one mechanism to improve antibody-binding affinity, the immediate impact of these observations was unclear because endogenous FcγR glycan composition, particularly of CD16a, was unknown. This deficit was largely due to technical limitations including small FcγR amounts in the body, multiple N-glycosylation sites, and challenges with purifying and analyzing integral membrane proteins. The sections below will evaluate the endogenous composition of FcγRs. These studies identified a mixture of compositions present on primary leukocytes, including those forms that promote strong interactions and some that bind with lower affinity to highlight the potential for FcγR improvements.

## Functional differences in FcγR alleles

Multiple variables contribute to FcγR variability including multiple alleles for some genes with distinct correlations to post-translational processing and antibody binding. Two predominant CD32a alleles encode either an R131 or H131 allotype; the latter exhibits greater IgG1-binding affinity (1). Two major CD16a alleles encode either a tighter-binding V158 allotype or the weaker F158 as discussed in the previous section. Minor CD16a forms include H48 (8%) or R48 (6%) in place of the more common L48 (86%) (32). Three CD16b alleles, NA1, NA2, or SH, are expressed as glycosylphosphatidylinositol-anchored proteins after proteolysis during expression, displaying negligible differences in antibody-binding affinity (1). Variability in major and minor FcγR alleles coupled with gene copy-number differences contribute to a highly dynamic and variable landscape of antibody-binding capability at the surface of an FcγR-expressing cell (27, 33). The nature and impact of these mixed receptor communities is unknown, but there is a potential for FcγR allotypes to interact in heretofore unpredicted ways, increasing the functional heterogeneity of the FcγR-expressing leukocytes from individual donors.

The high serum antibody concentration likewise impacts FcγRs at the cell surface. The *in vitro* equilibrium dissociation constants for IgG1 (1 nM–20 μM) are much lower than those of the IgG1 concentration in the serum (33–100 μM), suggesting all receptors are mostly to almost completely bound by antibodies in the serum. How do ligated receptors then bind immune complexes? Dissociation rates on the order of a second to minutes predict a reasonable level of ligand turnover for the “low-affinity” receptors that must first dissociate to bind immune complexes. Because the immune complexes have a higher affinity for the cell surface because of avidity effects, immune complex binding is favored. CD64 is a notable “high-affinity” FcγR that displays a very slow dissociation rate with a t_1/2_ ∼ 30 min although it is unclear how the slow kinetics impact cell activation. One benefit of this slow dissociation rate is that engineered CD64+ cells complexed with an antibody before infusion might retain the antibody for long enough to affect an antibody-mediated response in the body after infusion (34).

Antibodies are likewise glycoproteins with a distribution of glycoforms in the serum that provides a distribution of FcγR-binding affinities. Primary human NK cells retained a high percentage of tight-binding IgG1 glycoforms on the surface, likely selecting the tightest binding ligand from the pool of IgG1 in serum that contains much lower levels of this specific antibody glycoform (33). The tight-binding IgG1 glycoforms lack a fucose modification to the N-linked N-acetylglucosamine residue (fucose is a red triangle in Fig. 2; (35)). This enrichment indicates NK cells are potentially prioritizing responses to targets identified by antigen-specific antibodies lacking fucose found in patients with immune thrombocytopenia, HIV, and dengue and a likelihood of similar responses in other diseases (36, 37, 38).

## Variable processing

In addition to different allotypes, post-translational processing introduces significant FcγR heterogeneity. Substantial advances in mass spectrometry including glycopeptide separations and analysis that allow a characterization of FcγRs from primary tissue highlight how each cell type specifically modifies each FcγR. The remainder of this section will review glycoproteomic characterizations of FcγRs purified from primary tissue that have only become available in the last 3 years to reveal forms present in the human body. These recent advances prove that individual cell type and environment lead to unique processing that is not recapitulated with recombinant expression systems. These differences include a much higher degree of sialylation and hybrid forms, as well as substantial branch elongation (11). This critical observation underscores the importance of using primary cells because it is impossible to discover novel functional roles for individual N-glycans if the endogenous glycoforms are not present *in vitro*.

Characterizing endogenous FcγRs, as discussed below, identified individual substrates present on cells that potentially impact cell function. In some cases, clear connections are already available, including the connection of CD32a N-glycans and inhibitory *cis*-interactions with Mac1 or the presence of high-affinity CD16a glycoforms on NK cells but not monocytes. There are many other features that remain to be explored, including extended complex-type branching that may serve as docking sites for cross-linking proteins at the cell surface. It is likely that many of these modifications will provide unique features for targeted improvements to FcγR-mediated therapies.

### FcγRIIIa/CD16a

**Figure 3:**
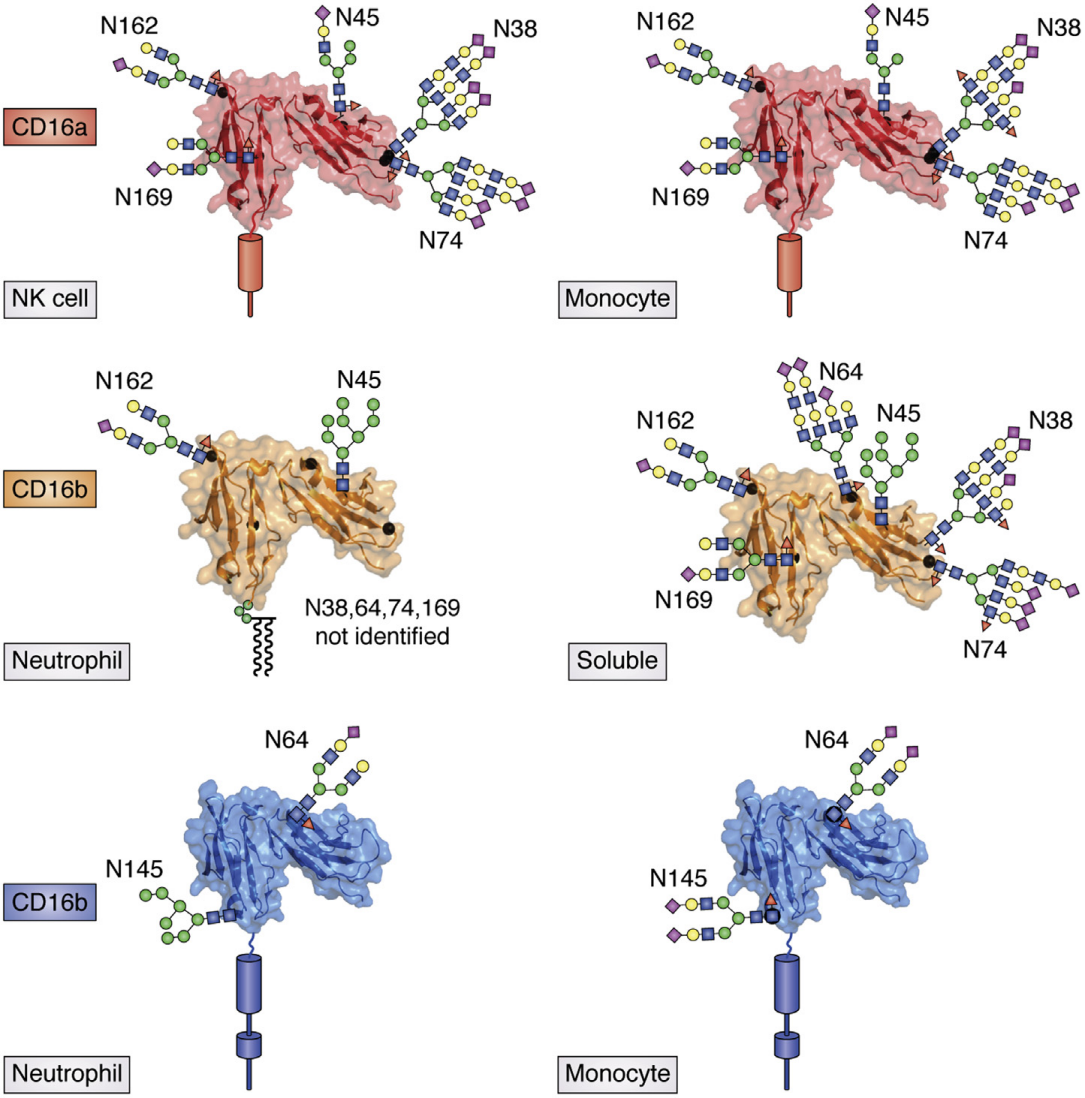
**The predominant Fc γ receptor glycoforms identified in the tissue or serum from healthy donors.** The N-glycans are scaled roughly to the appropriate size. The NK cell N-glycans increase the mass of the extracellular domain by 40%. The CD16 N45 glycan contributes to protein stability, the CD16a N162 glycan composition impacts antibody-binding affinity, and the CD32a N64 glycan inhibits antibody binding through *cis*-interactions on the neutrophil surface. N-glycan, glycans attached to Asn residues; NK, natural killer.

CD16a occupies a central role in many mAb therapies as the primary receptor initiating a cytotoxic cell–mediated response (39, 40). Furthermore, as noted above, CD16a processing affects antibody-binding affinity and potentially mAb efficacy; therefore, the characterization of CD16a processing is expected to inform mAb-based therapy development. It is not yet known, however, if CD16a glycoforms that bind tightly *in vitro* enhance cell activity or mAb efficacy. CD16a has five N-glycosylation sites, and composition at the N162 site directly impacts affinity *in vitro* (Fig. 3) (30).

#### N162

The composition of the N-glycan at N162, located near the IgG1 Fc-binding interface, influenced binding affinity *in vitro* with oligomannose forms promoting the tightest interactions (28). Primary NK cells from five healthy donors revealed a wide distribution of N-glycans found at this site, unlike monocytes with no apparent variability between donors (33, 41). One V158/F158 heterozygous NK cell donor expressed exclusively oligomannose forms at N162 on the V158 allotype, although the predominant glycan found across all NK cell donors was a core-fucosylated complex-type structure with two branches and a single *N*-acetylneuraminic acid (Fig. 3). The other four NK cell donors expressed 5 to 40% of minimally processed hybrid- and oligomannose-type N-glycans at this site. If the *in vitro* affinity measurements represent binding *in vivo*, NK cells express CD16a with a range of antibody-binding affinities and cells with a higher proportion of tight-binding oligomannose N162 glycoforms potentially bind IgG1 with greater affinity. It is possible that protein processing is responsive to extracellular signals and cells have the ability to dynamically modulate receptor-binding affinity, although this hypothesis remains untested. Furthermore, N162 was the only site that showed substantial composition differences between donors.

#### N45

Characterizations of recombinantly expressed CD16a indicated a high proportion of oligomannose-type N-glycans, likely because of the formation of substantial contacts between sugars at this site and amino acid side chains (42, 43, 44). It was therefore surprising that N45 from primary NK cells contained predominantly hybrid-type N-glycans (27, 33). Monocytes showed a higher percentage of oligomannose forms, but hybrid types remained predominant (41). Hybrid forms are found on secreted proteins but are less common than complex or oligomannose types. This finding is supported by an earlier glycomics study showing a high percentage of hybrid N-glycans on CD16a from primary NK cells of three geriatric donors (28). It is likely that restricted processing is a product of intramolecular contacts and does not appear to impact antibody-binding affinity, stabilizing the protein.

A single amino acid substitution found in a small percentage of donors impacts N45 processing and is related to NK cell dysfunction. Roughly 8% of the population expresses CD16a H48, instead of the more common L48 allotype (32). Homozygosity of the H48-encoding allele is believed to be responsible for an NK cell–related immunodeficiency (45). N45 from NK cell CD16a H48 in a heterozygous donor revealed greater processing, unlike the comparable processing at the four other N-glycosylation sites (46). It is possible that these N45-glycan differences are attributable to H48 disrupting CD2 binding and exposing the N45 site for further glycan processing (45).

Another consistent theme of FcγR N45 N-glycans from primary cells is a high degree of capping at the termini of complex-type branches, in particular with N-acetylneuraminic acid.

#### N38 and N74

Two N-glycosylated loops opposite the antibody-binding site display the most highly processed sites on CD16a expressed by primary NK cells and monocytes (33, 41). These are predominantly tetra-antennary complex-type N-glycans (containing four branches) with four sialic acid residues plus the addition of N-acetyllactosamine (LacNAc) repeats that extend the complex-type branches (Fig. 2). LacNAc repeats are potent galectin ligands and are important for T-cell and B-cell function, although it is not clear how these features impact NK cells and monocytes (47, 48). Monocytes expressed glycans at these sites with slightly less N-acetylneuraminic acid and a greater degree of branch fucosylation that may bind to different crosslinking factors.

#### N169

Comparable to N162, the N169 site contained predominantly a complex-type biantennary N-glycan with a single N-acetylneuraminic acid; however, highly processed tetra-antennary N-glycans with LacNAc repeats appeared at low levels in NK cells and monocytes (33, 41). It is unclear why N38 and N74 are processed to a high extent but N169 and N162 experience less processing. N162 forms observable contacts with the protein surface; however, these are markedly weaker than those formed by N45; N169 does not appear restricted (43). It is likewise unclear how N169 glycan composition impacts CD16a properties as it is dispensable for antibody binding (30).

### FcγRIIIb/CD16b

CD16b is highly homologous to CD16a at the amino acid level and accordingly shares many features with CD16a (Fig. 3). CD16b results from a gene duplication in humans after the split with chimps and is highly expressed as a glycosylphosphatidylinositol-anchored protein on neutrophils. CD16b is highly similar to CD16a at the amino acid level, with only a handful of differences in the extracellular domain when compared with CD16a in the sequence of the extracellular domain. There are, however, a few notable differences as discussed below in addition to two common allotypes: the CD16b NA2 and SH allotypes include an additional N-glycosylation site at N64 (six total sites), and the NA1 allele is not glycosylated at N45 because of a S47N substitution (four sites).

#### N38 and N74

N38 and N74 showed high levels of processing with branch sialylation and LacNAc repeats comparable with CD16a (49, 50).

#### N45

The CD16b NA2 N45 glycosylation site contains predominantly oligomannose-type N-glycans identified in contrast to the hybrid forms found on CD16a, although two reports differ in the proportion. One study using CD16b isolated from serum reported only oligomannose forms (49), whereas two others reported 80 to 90% oligomannose forms from neutrophils (27, 50). One report also identified lower percentages of oligomannose forms in soluble CD16 (40–60%) likely because of the presence of both CD16a and CD16b in the solution (27). Donor differences possibly explain the incongruent observations. It is unclear if cell- or protein-specific factors contribute to the significant processing differences when compared with the CD16a N45-glycan. Hetero-oligomers formed in the Golgi would limit processing of glycans buried during complexation, and glycan processing is a sensitive reporter of surface-exposed epitopes.

#### N64

Yagi *et al.* (49) reported high levels of N64 glycan processing for the soluble form of the NA2 allele, comparable with N38 and N74 but with less fucosylation and sialylation in contrast to Washburn *et al.* and Wojcik *et al.* who noted limited glycan occupancy at this site (27, 50, 51). It is unclear if N64 glycosylation impacts CD16b function.

#### N162

The primary CD16b N162-glycan was a core-fucosylated complex-type structure with two branches and a single α2-6 *N*-acetylneuraminic acid, consistent with CD16a, although no evidence for less-processed hybrid or oligomannose forms was found and one report analyzed material from 50 donors (27, 49, 50). The comparison with CD16a is notable because the IgG1 Fc binding affinity for the CD16b NA2 allotype, despite a high sequence identify to CD16a, is not affected by changing its N-glycan composition, unlike CD16a (30). Interestingly, the N-glycan sensitivity was introduced to CD16b NA2 through a D129G substitution, based on the residue found in CD16a that also increased affinity (31). Thus, a mechanism to modulate CD16a affinity that is specifically preserved does not appear to be present for CD16b. Finally, N162 processing on the NA1 allotype showed greater processing, potentially because of the loss of N45 glycosylation that serves to stabilize the two extracellular domains (27, 43). It is unclear if this increased processing affects function, but it is possible that adding sialic acid residues reduces antibody-binding affinity (26).

#### N169

This N-glycan shows substantial processing with a high degree of complex-type forms but less branching than glycans at N38 and N74 (49, 50).

### FcγRIIa/CD32a

CD32a contains two N-glycosylation sites, and both sites are occupied at high levels. A sialylated N64 glycan is required to interact with Mac1 and inhibits CD32a on the neutrophil surface (51).

#### N64

Two separate studies identified predominantly sialylated complex-type N64-glycans with a monosialylated form predominant on neutrophils and a disialylated form on monocytes (41, 51) (Fig. 3). These reports identified biantennary and triantennary structures on both cell types with some occupancy of oligomannose forms on neutrophils and hybrid forms on monocytes. In either case, sialylated N-glycans are present to form interactions with Mac1.

#### N145

N145 showed comparable though slightly less processing than the N64 site on CD32a isolated from monocytes. These sites contain the same predominant sialylated biantennary glycans but a few more hybrid forms at lower abundance were present on N145. The N145 site of CD32a isolated from neutrophils, however, showed dramatically less processing with >75% oligomannose and hybrid forms. This difference in processing by two different cell types may indicate the presence of an additional factor in neutrophils that prevents N145 processing, comparable with the association of CD16a and CD2 in NK cells. These differences again suggest the possibility of cell type–specific functional diversification through N-glycan processing.

### Soluble FcγRs

Activated leukocytes shed the receptor extracellular domains in the serum after proteolysis, with the exception of CD64. Soluble forms modulate multiple processes although it is not clear whether soluble receptor forms are simply inert products of activated cells or adopt specific anti-inflammatory roles (11). In principle, soluble receptors could compete with soluble antibodies and prevent FcγR-mediated cell activation, although antibodies greatly outnumber receptors in the peripheral compartment.

On NK cell activation induced by an antibody-coated target or artificially through a protein kinase C agonist, CD16a is shed after ADAM17-mediated proteolysis at S197 (52). Shedding likely increases serial engagement of target cells (one NK cell killing more than one target cell) and increases NK cell survival (53). ADAM17 is likewise responsible for shedding CD16b in neutrophils (54).

### Conclusions from FcγR processing

Characterizing the products of endogenous FcγR processing identified the substrates to define the role of these individual modifications in receptor function and cell activity. Links between composition and function are known for sialylated CD32a N64 N-glycans from neutrophils that mediate Mac-1 interactions, and there is a possible role of CD16a N162 glycoforms in tuning antibody-binding affinity. The function of many other distinct features is not known, including LacNAc repeats on CD16 N38 and N74 that are found on at least 3 cell types. This compositional conservation strongly suggests a role in FcγR function. It is also possible that individual modifications, including branch fucosylation, impact complexes formed by receptors. Any functionally relevant modification has the potential to impact FcγR-dependent therapies, and it is likely that multiple critical glycan-mediated processes remain undiscovered. It is also important to note donor-, cell type-, and FcγR-specific differences in processing. It is clear that each site of each FcγR exists in an individual environment, and the individual processing must be studied at this level of resolution.

## FcγR engineering

Antibodies have been the focus of many engineering efforts likely because of the rapidly deployable and highly generalizable nature of mAbs with relatively little attention paid to FcγRs themselves (55). Although FcγRs provide useful modules for chimeric protein design, these designs generally do not enhance FcγR functionality and, with one exception, will not be discussed here (a few examples are included (56, 57, 58, 59)). These early FcγR engineering reports provide a strong justification for further engineering to increase affinity and increase the activity of engineered leukocytes.

Engineering NK cells to express the stronger antibody binding CD16a V158 allotype benefits cancer treatment. CD16a V158 was reintroduced to cultured NK92 cells, originally derived from a 50-year-old patient but lack CD16 expression and antibody-mediated cytotoxicity (60, 61). After infusion, these cells showed greater efficacy toward the tumor than the patient’s endogenous NK cells. It remains to be seen if generating greater CD16a expression levels will lead to further increases in efficacy.

FcγR engineering to limit shedding is designed to improve cell function. The thought is that leukocytes that do not shed FcγRs will maintain an ability to engage new targets. CD16a is shed after ADAM17-mediated proteolysis at S197 (52). The CD16a S197P variant prevented proteolysis and shedding while improving the antitumor activity of human induced pluripotent stem cell-derived NK cells (62). Negative aspects of eliminating shedding include decreased serial engagement of target cells (one NK cell killing more than one target cell) and decreased NK cell survival (53).

One chimeric FcγR construct combined the antibody-binding affinity of the CD64 extracellular domain with the CD16a signaling interactions (34). This construct enhanced antibody-dependent cytotoxicity in NK92 and human induced pluripotent stem cell-derived NK cells. These cells also exhibited an increase in natural cytotoxicity, which is somewhat surprising, given a role for the CD16a extracellular domain in natural cytotoxicity (45). These cells likewise retained mAb to a greater extent after washes that may prove beneficial for *ex vivo* ligation followed by infiltration. It is unclear, however, if these cells retain the activity in the serum that may prevent immune complex binding by occupying the CD64 extracellular antibody-binding domain.

## Engineering potential

Improving the FcγR function may be achieved by targeting the polypeptide or carbohydrate components. The recent developments in lymphocyte engineering described above provide a suitable platform to deploy engineered receptors.

The potency of carbohydrate manipulation as a strategy to improve biopharmaceutical efficacy is demonstrated by afucosylated mAbs (including Gazyva, discussed above) showing improved cytotoxicity and efficacy. Multiple glycoengineered antibodies are in various stages of development and likely represent the next generation of mAbs. FcγR glycoengineering would require targeted host cell manipulation of the known glycosyltransferase and glycosylhydrolase enzymes. Potential caveats include changing cell glycosylation to modify only the FcγR of interest without impacting other glycan-related processes. A worthy design goal for CD16a and CD32a includes reducing/eliminating sialylation to increase antibody-binding affinity in CD16a and prevent Mac-1–mediated inhibition of CD32a on neutrophils. Alternative approaches may include modifying the CD16a N162-glycan composition as oligomannose forms bind antibodies with greater affinity. Alternative, truncated forms at this site are possible through engineered site-specific glycosylhydrolases (63). Truncating the CD16a N162-glycan to a single N-acetylglucosamine residue promises to improve affinity to a greater extent than an oligomannose N-glycan; *in vitro* studies demonstrated that CD16a with truncated N-glycans bound IgG1 Fc tighter than CD16a with unmodified oligomannose N-glycans (44).

Many of the challenges associated with glycoengineering leukocytes to express customized FcγRs may be alleviated by engineering extracellular domains for immune system modulation as soluble, serum-borne fragments. These proteins are expected to dampen antibody-mediated mechanisms as is proposed for their natural soluble counterparts.

Engineering the polypeptide fraction of FcγRs represents a substantial challenge, but one with a considerable payoff considering the impact of engineered IgG Fcs that were developed from computational design and directed evolution (64). FcγRs could be engineered to enhance favorable properties, including increased antibody-binding affinity or greater clustering, residence time, and so forth. One challenge of directed evolution techniques is preparing proteins with appropriate processing. Although yeast strains producing mammalian glycans are available, expression yield is generally low and the glycan repertoires are limited (65). Surface display using a mammalian expression host may provide a more tractable engineering platform (66, 67, 68, 69).

## Conclusions

**Figure 4:**
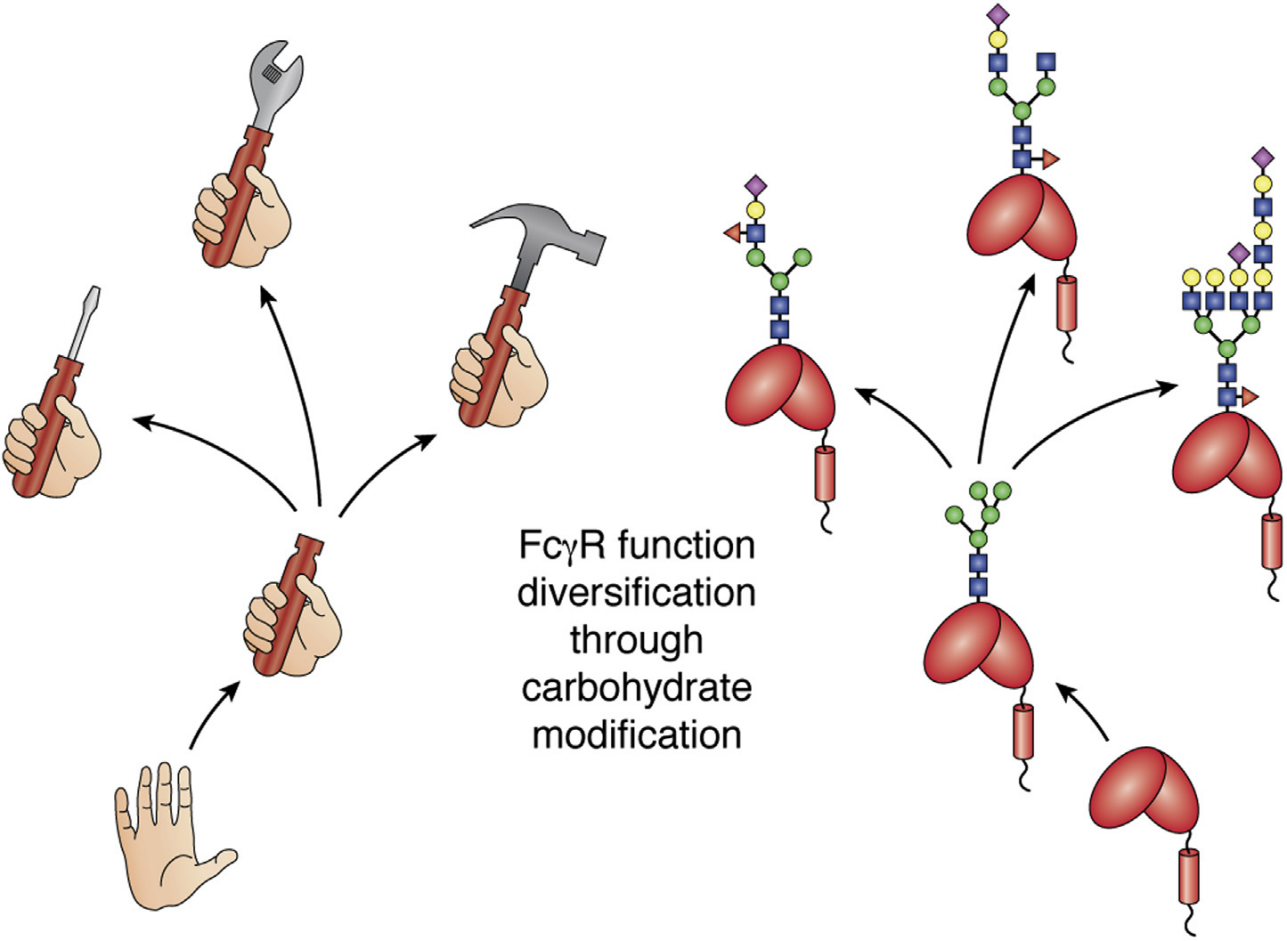
**Extensive carbohydrate modifications provide FcγRs with distinct functions.** Some enhance antibody-binding affinity, some repress activity, and some likely bind cell matrix proteins. FcγRs, Fc γ receptors.

The FcγRs promote or suppress immune responses and represent an important motif for engineering to increase the efficacy of antibody-mediated therapies. These surface cell receptors are highly heterogeneous, resulting from different genes, alleles, and differential processing. Processing in the form of extensive carbohydrate additions provides different functions to each receptor, although this area is only lightly explored and there is a distinct possibility that multiple functions remain undefined (Fig. 4). In addition, selecting/enhancing FcγR forms with superior properties from the pool available in the body or engineering these receptors to increase function promises new treatments to many diseases.

## Conflict of interest

The author declares that they have no conflicts of interest with the contents of this article.
